# Prognostic value of CYFRA 21 − 1 and Ki67 in advanced NSCLC patients with wild-type EGFR

**DOI:** 10.1186/s12885-023-10767-9

**Published:** 2023-03-31

**Authors:** Tao Li, Qi Xie, Yang-Yang Fang, Yi Sun, Xiao Ming Wang, Zhu Luo, Gui-Ling Yan, Jian-Bo He, Xiao-Qun Zheng

**Affiliations:** 1 Department of Laboratory Medicine, The Second Affiliated and Yuying Children’s Hospital of Wenzhou Medical University, Wenzhou, China; 2 School of Laboratory Medicine and Life Sciences, The Key Laboratory of Laboratory Medicine, Wenzhou Medical University, Ministry of Education of China, Wenzhou, China; 3 Department of Respiratory Medicine, The Second Affiliated and Yuying Children’s Hospital of Wenzhou Medical University, Wenzhou, China

**Keywords:** CYFRA 21 − 1, Ki67, NSCLC, Wild-type EGFR, Prognosis

## Abstract

**Background:**

The prognostic value of cytokeratin 19 fragment (CYFRA 21 − 1) and Ki67 in advanced non-small cell lung cancer (NSCLC) patients with wild-type epidermal growth factor receptor (EGFR) remains to be explored.

**Methods:**

In this study, 983 primary NSCLC patients from January 2016 to December 2019 were retrospectively reviewed. Finally, 117 advanced NSCLC patients with wild-type EGFR and 37 patients with EGFR mutation were included and prognostic value of CYFRA 21 − 1 and Ki67 were also identified.

**Results:**

The patients age, smoking history and the Eastern Corporative Oncology Group (ECOG) performance scores were significantly different between CYFRA21-1 positive and negative groups (*p* < 0.05), while no significant differences were found in Ki67 high and low groups. The results of over survival (OS) demonstrated that patients with CYFRA21-1 positive had markedly shorter survival time than CYFRA21-1 negative (*p* < 0.001, For whole cohorts; *p* = 0.002, For wild-type EGFR). Besides, patients with wild-type EGFR also had shorter survival times than Ki67 high group. Moreover, In CYFRA 21 − 1 positive group, patients with Ki67 high had obviously shorter survival time compared to patients with Ki67 low (median: 24vs23.5 months; *p* = 0.048). However, Ki67 could not be used as an adverse risk factor for patients with EGFR mutation. Multivariate cox analysis showed that age (HR, 1.031; 95%CI, 1.003 ~ 1.006; *p* = 0.028), Histopathology (HR, 1.760; 95%CI,1.152 ~ 2.690; *p* = 0.009), CYFRA 21 − 1 (HR, 2.304; 95%CI,1.224 ~ 4.335; *p* = 0.01) and Ki67 (HR, 2.130; 95%CI,1.242 ~ 3.652; *p* = 0.006) served as independent prognostic risk factor for advanced NSCLC patients.

**Conclusions:**

Our finding indicated that CYFRA 21 − 1 was an independent prognostic factor for advanced NSCLC patients and Ki67 status could be a risk stratification marker for CYFRA 21 − 1 positive NSCLC patients with wild-type EGFR.

**Supplementary Information:**

The online version contains supplementary material available at 10.1186/s12885-023-10767-9.

## Introduction

Non-small cell lung cancer (NSCLC) is the predominant subtype of lung cancer which is still the leading cause of cancer-related deaths in the world [1]. Due to the limitation of tumor markers for diagnosis in the early stage of cancer, more than half of patients were diagnosed in advanced stage at the first time of admission. The comprehensive treatment model based on chemotherapy is still the main treatment strategy for NSCLC patients [2]. Although the application of various tyrosine kinase inhibitors (TKIs) could prolong the survival time of NSCLC patients who had epidermal growth factor receptor (EGFR) mutation, patients with wild-type EGFR in advanced NSCLC are still not optimistic [3]. Therefore, it is important to find reliable markers or evaluation criteria for early diagnosis or prognosis prediction for NSCLC patients.

Multiple prognostic factors, such as patient’s age, histological type, TNM stage, progression-free survival (PFS), and tumor marker levels, were reported in NSCLC patients [4–7]. Among them, tumor markers were widely used in the diagnosis and prognosis monitoring of NSCLC, Carcinoembryonic Antigen (CEA) and CYFRA 21 − 1 were considered the most sensitive serum marker for NSCLC diagnosis and prognosis prediction [5, 8, 9]. CYFRA 21 − 1 is a fragment of cytokeratin 19, a protein found in the cytoskeleton of epithelial cells, including bronchial epithelium. It is released at increased levels in tumors of epithelial origin such as lung cancer. Mild elevations can also be found in benign lung diseases such as tuberculosis, chronic obstructive pulmonary disease, acute lung infections, and fibrosis [10, 11]. Besides, Yao and colleagues demonstrated that CYFRA 21 − 1 and Squamous Cell Carcinoma Antigen (SCC) could be applied to predict the EGFR mutation status in lung cancer [12]. Ki67 is well known as a nuclear antigen related to cell proliferation and closely related to tumor proliferation, invasion, metastasis, and prognosis [13]. For solid tumors, such as breast cancer, Ki67 is negatively correlated with the patient’s prognosis [14]. However, the clinical value of Ki67 in non-small cell lung cancer is still being explored. Some studies demonstrated that Ki67 was inversely correlated with the prognosis of patients with resected NSCLC [15, 16]. However, another study demonstrated that Ki67 might not associated with prognosis and the better strategy was combing with other molecules for prognosis evaluation [17]. Therefore, whether the combination of CYFRA 21 − 1 with Ki67 for evaluating the prognosis in advanced NSCLC patients with wild-type EGFR is reliable or not is unclear.

In the present study, we retrospectively collected clinical and follow-up records on patients diagnosed with advanced NSCLC in our hospital and further analyzed the prognostic value of CYFRA 21 − 1 and Ki67 between 117 advanced NSCLC patients with wild-type EGFR and 37 advanced NSCLC patients with EGFR mutation.

## Patients and methods

### Study design and patient cohort

**Fig. 1:**
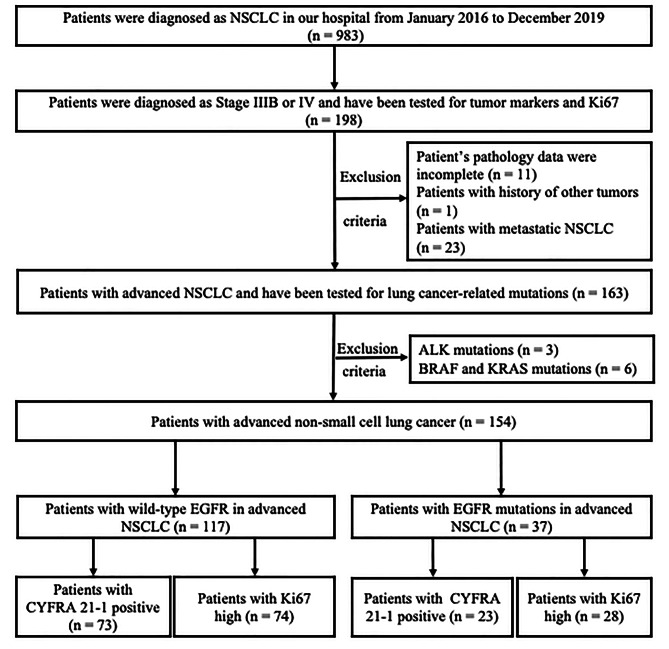
Study design and algorithm of patient selection

983 patients with primary NSCLC who were diagnosed by histology or cytology pathology from January 2016 to December 2019 at the Second Affiliated Hospital & Yuying Children’s Hospital of Wenzhou Medical University were retrospectively reviewed. Finally, 198 patients were diagnosed with primary IIIB or IV NSCLC. Among these 198 patients, 44 patients were excluded for the following reasons: 11 patients were excluded because they did not have complete pathology data; 1 patient was excluded because he had a history of other tumors; 23 patients were excluded because they were diagnosed metastatic NSCLC; 3 patients with ALK-positive and 6 patients with BRAF and KRAS mutations were also excluded. The study design and algorithm of patient inclusion were detailed in Fig. 1.

The clinical characteristics including age, gender, smoking history, family history of tumors, TNM stage, and histopathology were recorded. We defined nonsmokers as never-smokers or those who smoked less than 100 cigarettes in their lifetime and others were classified as ever-smokers. Four tumor markers of patients were tested in our laboratory including CEA, SCC, CYFRA 21 − 1, and Neuron Specific Enolase (NSE). The pathological types were classified as adenocarcinoma, squamous cell carcinoma, and others according to the latest WHO classification criteria for lung cancer. Moreover, the lung cancer was staged according to the TNM stage criteria of the International Association for the Study of Lung Cancer [18, 19]. The study was approved by the Research Ethics Boards of the Second Affiliated Hospital & Yuying Children’s Hospital of Wenzhou Medical University (2021-K-364-01). Informed consent was obtained from all patients included in the study. All experiments conformed to the Declaration of Helsinki. The detailed clinical information of the patients showed in Table 1 and Table 2.

**Table 1:** Clinical and laboratory characteristics for advanced NSCLC patients with wild-type EGFR

Variables	CYFRA 21 − 1		*p*	Ki67		*p*
	Positive (n = 73)	Negative (n = 44)		High (n = 74)	Low (n = 43)	
Gender			0.07			0.057
Male (n, %)	56 (76.71)	27 (61.36)		57 (77.02)	26 (60.47)	
Female (n, %)	17 (23.29)	17 (38.64)		17 (22.98)	17 (39.53)	
Age (years)			< 0.001			0.563
≥ 60 (n, %)	59 (73.8)	21 (47.73)		52 (70.27)	28 (65.12)	
< 60 (n, %)	14 (37.8)	23 (52.27)		22 (29.73)	15 (34.88)	
History of smoking			0.021			0.544
Never (n, %)	41 (56.16)	15 (34.09)		37 (50.00)	19 (44.19)	
Ever (n, %)	32 (43.84)	29 (65.91)		37 (50.00)	24 (55.81	
Family history of tumors (n, %)			0.268			0.695
Yes (n, %)	2 (2.74)	0 (0)		1 (1.35)	1 (2.33)	
No (n, %)	71 (97.26)	44 (100.00)		73 (98.65)	42 (97.67)	
ECOG score			0.012			0.838
0–2 (n, %)	57 (78.08)	42 (95.45)		63 (85.14)	36 (83.72)	
3–4 (n, %)	16 (21.92)	2 (4.55)		11 (14.86)	7 (16.28)	
Histology						
Adenocarcinoma (n, %)	35 (47.95)	30 (68.18)		39 (52.70)	27 (62.79)	
Squamous cell carcinoma	29 (39.73)	6 (12.64)		25 (33.78)	12 (27.91)	
Others (n, %)	9 (12.32)	8 (18,18)		10 (13.52)	4 (9.30)	
TNM Stage			0.976			0.332
IIIB (n, %)	28 (38.36)	17 (38.64)		26 (35.14)	19 (44.19)	
IV (n, %)	45 (61.64)	27 (61.36)		48 (64.86)	24 (55.81)	
CEA			0.55			0.431
Positive (n, %)	34 (46.58)	23 (52.27)		34 (45.95)	23 (53.49)	
Negative (n, %)	39 (53.42)	21 (47.73)		40 (54.05)	20 (46.51)	
NSE			0.007			0.588
Positive (n, %)	31 (42.47)	8 (18.19)		26 (36.49)	24 (55.81)	
Negative (n, %)	41 (57.53)	36 (81.81)		47 (63.51)	19 (44.19)	
SCC			0.903			0.205
Positive (n, %)	11 (15.07)	7 (21.2)		9 (12.16)	9 (20.93)	
Negative (n, %)	62 (84.93)	37 (44.0)		65 (87.84)	34 (79.07)	
TTF-1			0.022			0.492
Positive (n, %)	56 (76.71)	41 (93.18)		60 (81.08)	37 (86.05)	
Negative (n, %)	17 (23.29)	3 (6.82)		14 (18.92)	6 (13.95)	
CK-7			0.153			0.674
Positive (n, %)	53 (72.60)	37 (84.09)		56 (75.68)	34 (79.07)	
Negative (n, %)	20 (27.40)	7 (15.91)		18 (24.32)	9 (20.93)	
Line of therapy			0.965			0.497
1st	63	38		66	35	
2nd	6	4		5	5	
>2nd	4	2		3	3	

Note: Carcinoembryonic Antigen, CEA; Neuron Specific Enolase, NSE; Squamous Cell Carcinoma Antigen, SCC; Soluble Fragment of Cytokeratin 19, CYFRA 21 − 1; Thyroid transcription factor-1, TTF-1; cytokeratin-7, CK-7

**Table 2:** Clinical characteristics for 37 advanced NSCLC patients with EGFR mutation

Variables	Patients with EGFR mutations in advanced NSCLC
Gender, Female (n, %)	20 (54.05)
Age, ≥ 60 (n, %)	26 (70.27)
History of smoking, ever (n, %)	29 (78.38)
Family history of tumors, yes (n, %)	0 (0)
ECOG score, 0–2 (n, %)	34 (91.89)
Histology, Adenocarcinoma (n, %)	37 (100)
TNM Stage, IV (n, %)	33 (89.19)
CYFRA 21 − 1, Positive (n, %)	24 (64.86)
CEA, Positive (n, %)	27 (72.97)
NSE, Positive (n, %)	13 (35.14)
SCC, Positive (n, %)	4 (10.81)
Ki67, Positive (n, %)	28 (75.68)
TTF-1, Positive (n, %)	32 (86.49)
CK-7, Positive (n, %)	31 (83.78)
TKIs treatment	
Gefitinib (n, %)	29 (78.38)
Others TKIs (n, %)	8 (21.62)
EGFR mutations	
Exon 19 (n, %)	18 (48.65)
Exon 20 (n, %)	3 (8.11)
Exon 19 and 20 (n, %)	4 (10.81)
Exon 21 (n, %)	10 (27.03)
Exon 21 and 20 (n, %)	1 (2.70)
Exon 20 and 18 (n, %)	1 (2.70)

Note: Carcinoembryonic Antigen, CEA; Neuron Specific Enolase, NSE; Squamous Cell Carcinoma Antigen, SCC; Soluble Fragment of Cytokeratin 19, CYFRA 21 − 1; Thyroid transcription factor-1, TTF-1; cytokeratin-7, CK-7

Follow-up information was documented from January 2016 to May 2021. Overall survival (OS) was calculated from the date of diagnosis to the death or last follow-up Eventually, 102 cancer-related deaths were recorded which includes 25 stages IIIB and 77 stages IV patients, respectively.

### The measurement and analysis of serum tumor markers

The levels of four serum tumor markers were tested by the electrochemiluminescence method (Roche cobas 8000, Germany). Blood samples from all patients were obtained through peripheral venipuncture before anticancer treatment. The following thresholds were considered the upper limits of normal: CEA ≥ 4.7 mg/L, CYFRA 21 − 1 ≥ 3.3 ng/mL, SCC ≥ 1.5 mg/mL, NSE ≥ 16.3 ng/mL. Accordingly, tumor marker values above these thresholds were considered positive.

### Ki67 measurement and analysis

The expression of Ki67 in 117 NSCLC lesions was tested by the immunohistochemistry method (Fuzhou Maixin Company, Fujian, China). The criteria of Ki67 staining evaluation were as follows: Ki67 exists in the nucleus and the nuclei. Those stained with brownish yellow and brown are positive cells, while the staining intensity is irrespective. Ki67 negative (-): the number of Ki67 positive cells is less than 5% of the total cells; weakly positive (+): 5% ~ 25%; median positive (++): 25% ~ 50%; strong positive (+++): > 50%. (-) and (+) were defined as Ki67 low. (+) and (+++) were defined as Ki67 high.

Thyroid transcription factor-1 (TTF-1) and cytokeratin-7 (CK-7) were also detected by the immunohistochemistry method. The nuclei stained with brownish yellow > 25%.

### Mutation analysis

The total genomic DNA tumors were extracted using the TIANamp Genomic DNA kit (Tiangen Biotechnology Co., Ltd., Beijing, China) according to the manufacturer’s protocol. Quantitative mutation detection for *EGFR*, *ALK*, *BRAF*, and *KRAS* was carried out using ACCBio’s human gene mutation quantitative detection kit (ACCB Biotech Co., Ltd., Co., Beijing, China).

### Statistical analysis

Statistical analyses were performed using the software SPSS V. 26.0. (SPSS; Chicago, IL, USA). Categorical data were compared between groups using Fisher’s exact test or the chi-square test. The relationship between clinical features and overall survival (OS) was analyzed through univariate and multivariate analysis. The OS among each group was compared by Log-rank test. The prognostic factors for OS in advanced NSCLC patients with wild-type EGFR through multivariate cox analysis (including HR, 95%CI, and *p* value). A *p* value less than 0.05 was considered significant.

## Results

### Clinical and Laboratory characteristics for advanced NSCLC patients

Among 117 advanced NSCLC patients with wild-type EGFR, 73 patients were CYFRA 21 − 1 positive (62.39%; range 3.34 ~ 267.70 ng/mL) and 74 patients (63.24%) had high Ki67. Our study showed that patients age ≥ 60 years (*p* < 0.001), smoking history (*p* = 0.021), NSE (*p* = 0.007), ECOG scores (*p* = 0.012), and TTF-1 (*p* = 0.022) had significant differences between CYFRA 21 − 1 positive and negative groups, while no differences were observed in gender, family tumor history, SCC, Tumor stages, and CEA (Table 1). However, no differences between patients with Ki67 low or high were not observed in the above clinical and laboratory features (Table 1).

Among 37 advanced NSCLC patients with EGFR mutation, 23 patients were CYFRA 21 − 1 positive (62.16%; range 3.40 ~ 17.35 ng/mL); 28 patients had high Ki67 (75.68%); 27 patients were CEA positive (72.97%; range 5.610~**>**1000.00 mg/L); 4 patients were SCC positive (10.81%; range 1.60 ~ 1.74 ng/ml); 13 patients were NSE positive (35.14%; range 16.79 ~ 32.67 ng/mL); 32 patients were TTF-1 positive (86.49%), and CK-7 positive (83.78%). Besides, the most common EGFR mutation type were found in exon 19 and 21 (n = 18, 48.67%, for exon 19; n = 10, 27.02%, for exon 21) and 29 patients have selected gefitinib as first line treatment (Table 2).

### The relevance of CYFRA 21 − 1 and Ki67 to survival in advanced NSCLC patients

**Fig. 2:**
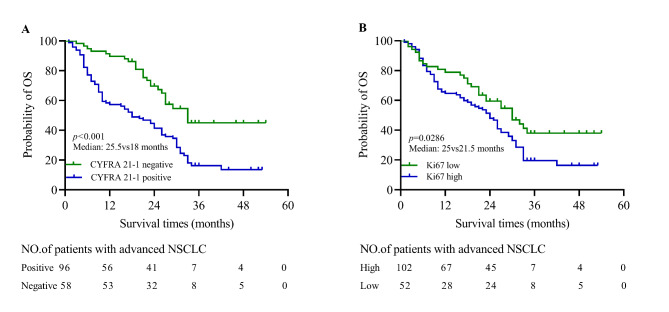
**The relevance of CYFRA 21 − 1 and Ki67 to survival in advanced NSCLC patients**. (**A**)The difference of prognosis between CYFRA 21 − 1 positive and negative groups (median: 25.5vs18 months, *p* < 0.001). (**B**)The difference of prognosis between low and high Ki67 groups (median: 25.5vs21.5 months, *p* = 0.0286)

For the whole cohort of 154 NSCLC patients, the median follow months was 23 months (range, 1 ~ 53 months). Kaplan-Meier survival curve showed that patients with CYFRA 21 − 1 positive have significantly shorter survival times than patients with CYFRA 21 − 1 negative (median: 18vs25.5 months, *p* < 0.001; Fig. 2A). Moreover, the survival difference was also observed in the Ki67 low and high groups (median: 25vs21.5 months, *p* = 0.0286; Fig. 2B).

**Fig. 3:**
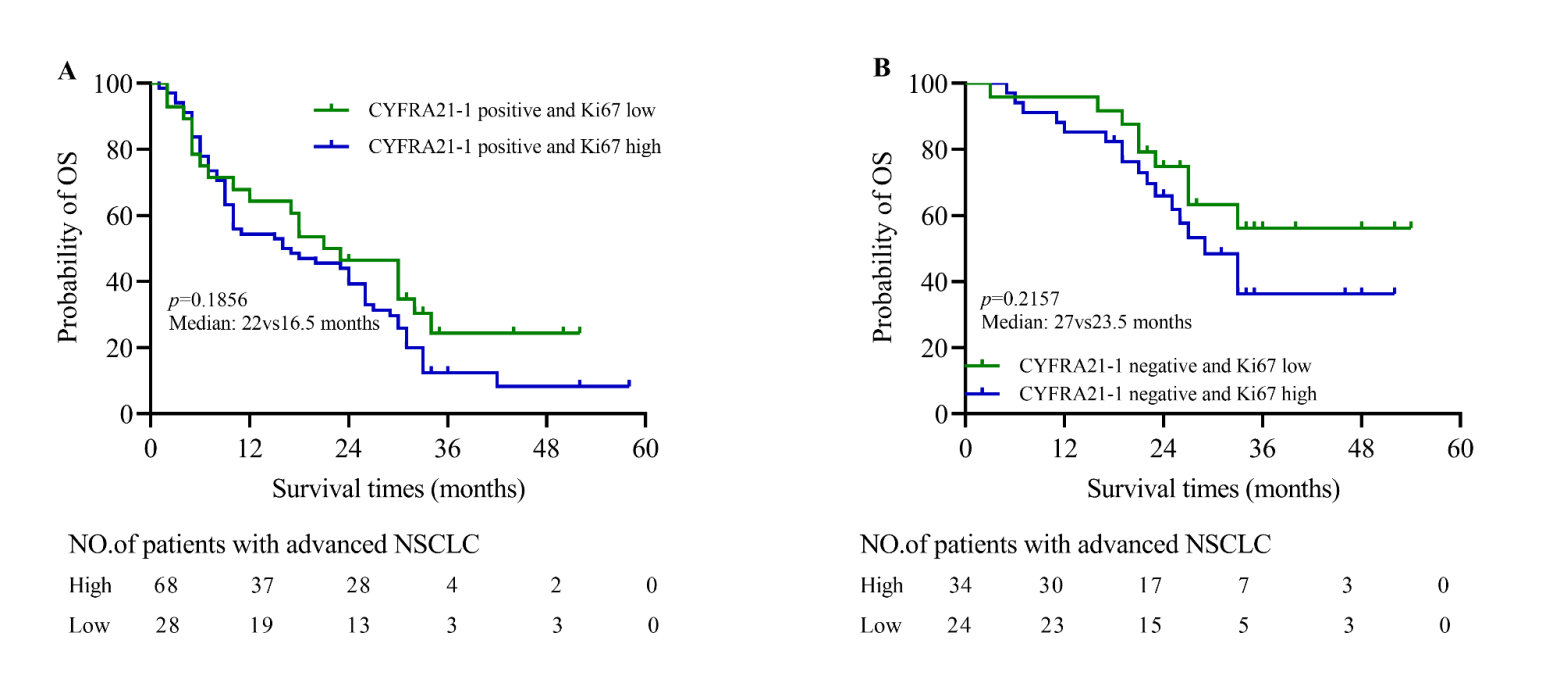
**The value of CYFRA 21 − 1 combined with Ki67 in predicting the prognosis of advanced NSCLC patients**. (**A**) Patients with CYFRA 21 − 1 positive and Ki67 high have not shorter survival times than patients with CYFRA 21 − 1 positive and Ki67 low (median: 22vs21.5 months, *p* = 0.1856). (**B**) Ki67 could also not use as a prognosis evaluation marker in CYFRA 21 − 1 negative group (median: 27vs23.5 months, *p* = 0.2157)

In the CYFRA 21 − 1 positive with Ki67 high or low groups, survival times of patients with CYFRA 21 − 1 positive and Ki67 high was not significantly lower than patients with CYFRA 21 − 1 positive and Ki67 low (median: 22vs16.5 months, *p* = 0.1856; Fig. 3A). Besides, in CYFRA 21 − 1 negative subgroup, Ki67 might not serve as a prediction factor (median: 27vs23.5 months, *p* = 0.2157, Fig. 3B).

### The relevance of CYFRA 21 − 1 and Ki67 to survival in advanced NSCLC patients with wild-type EGFR

**Fig. 4:**
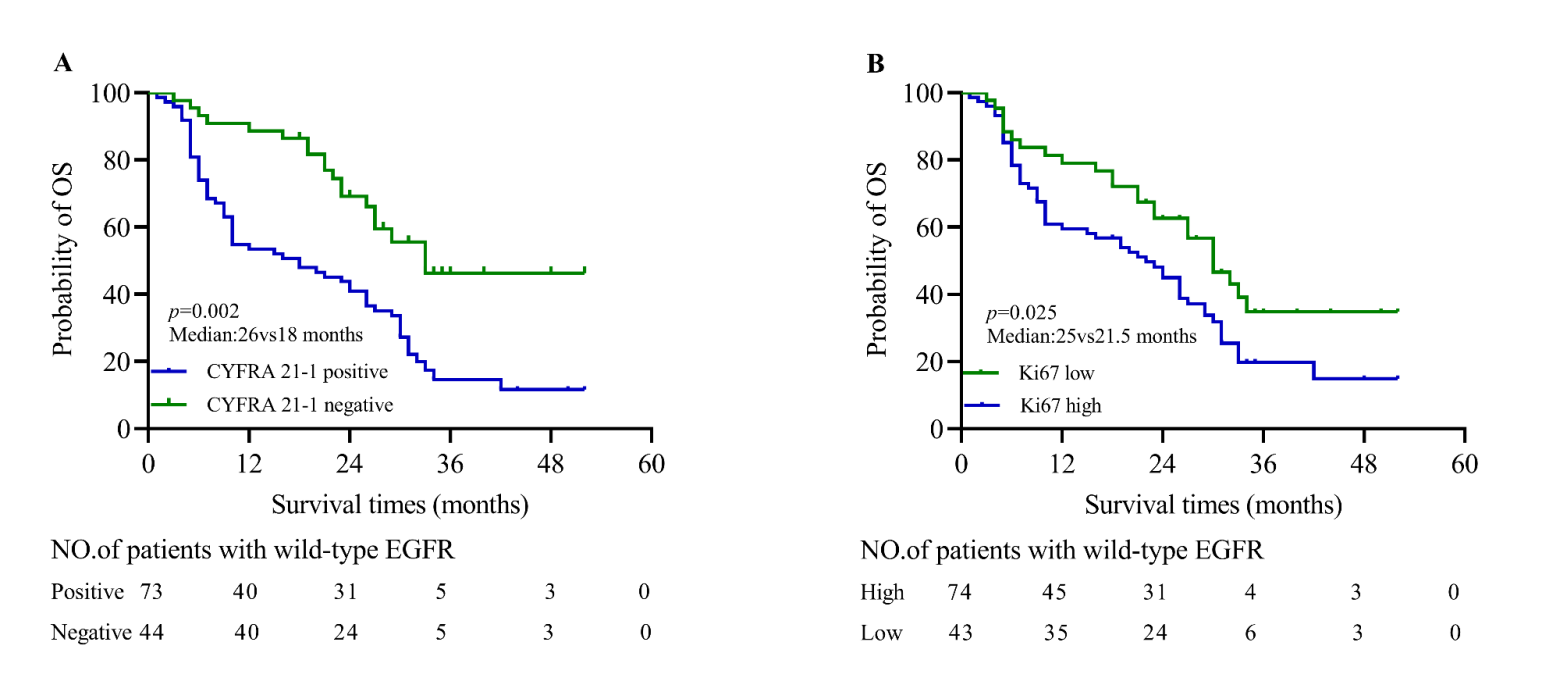
**The relevance of CYFRA 21 − 1 and Ki67 to survival in advanced NSCLC patients with wild-type EGFR.**. (**A**)The difference of prognosis between CYFRA 21 − 1 positive and negative groups (median: 26vs18 months, *p* = 0.002). (**B**)The difference between low and high Ki67 groups (median: 25vs21.5 months, *p* = 0.025)

Among the 117 NSCLC patients with wild-type EGFR, the median follow times were 17 months (range, 1 ~ 52). Kaplan-Meier survival curve showed that patients with CYFRA 21 − 1 positive have significantly shorter survival times than patients with CYFRA 21 − 1 negative (median: 26vs18 months, *p* = 0.002; Fig. 4A). Besides, the difference was also observed in Ki67 low and high groups (median: 26vs20 months, *p* = 0.025; Fig. 4B).

**Fig. 5:**
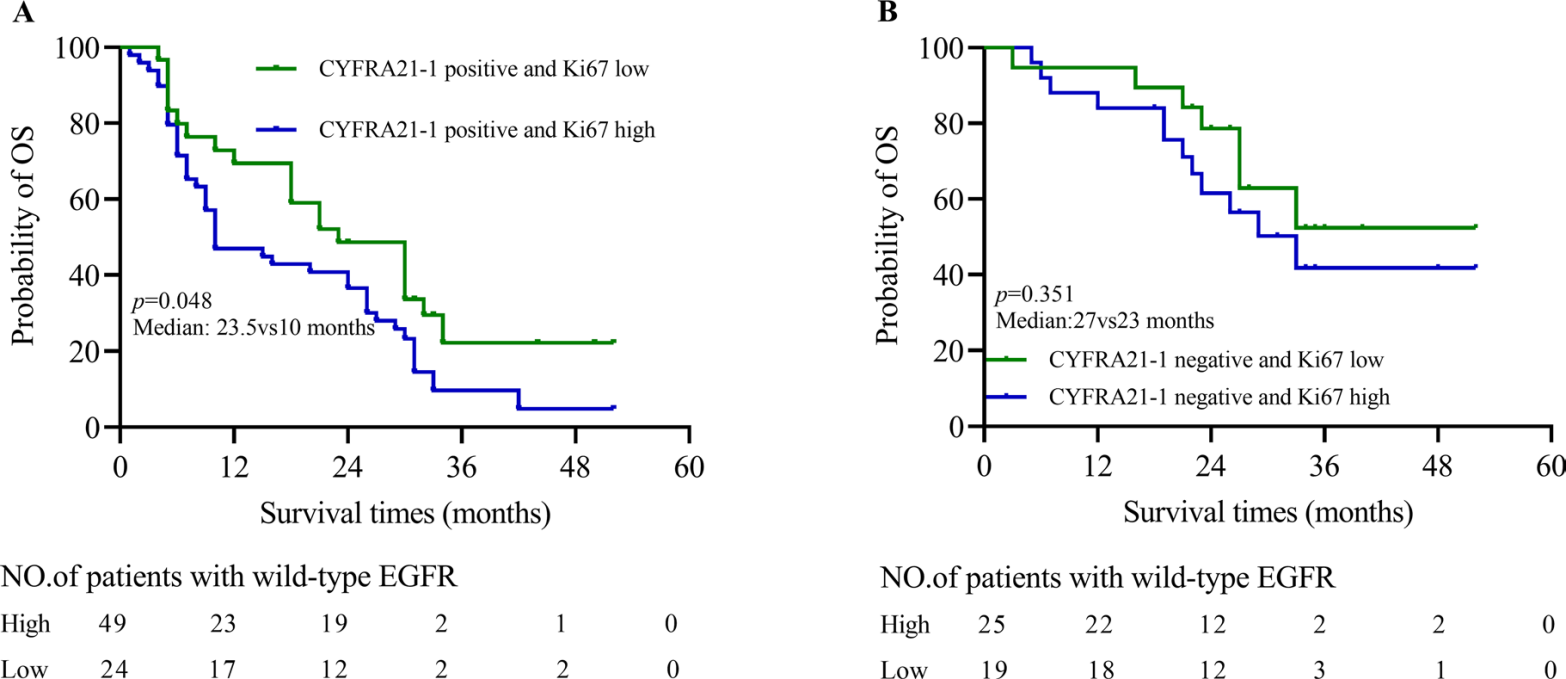
**The value of CYFRA 21 − 1 combined with Ki67 in predicting the prognosis of patients with wild-type EGFR in advanced NSCLC.**. (**A**) Patients with CYFRA 21 − 1 positive and Ki67 high have shorter survival times than patients with CYFRA 21 − 1 positive and Ki67 low (median: 23.5vs10 months, *p* = 0.048). (**B**) Ki67 could not use as a prognosis evaluation marker in CYFRA 21 − 1 negative group (median: 27vs23 months, *p* = 0.3510)

In the CYFRA 21 − 1 positive with high or low Ki67 groups, survival times of patients with CYFRA 21 − 1 positive and Ki67 high (n = 49; range 1 ~ 52 months) were significantly lower than patients with CYFRA 21 − 1 positive and Ki67 low (n = 24; median: 10vs23.5 months, *p* = 0.048; Fig. 5A). However, in CYFRA 21 − 1 negative subgroup, the Ki67 could not use as a prediction factor (median: 27vs23 months, *p* = 0.3510, Fig. 5B).

### The relevance of CYFRA 21 − 1 and Ki67 to survival in advanced NSCLC patients with EGFR mutation

**Fig. 6:**
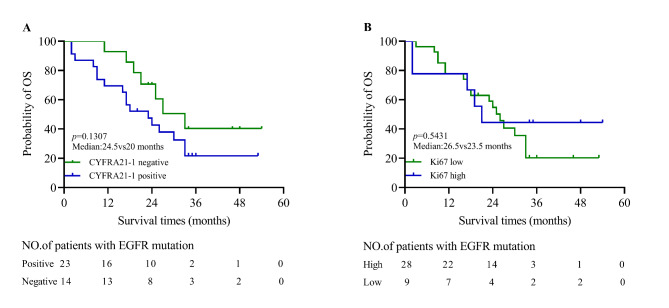
**The value of CYFRA 21 − 1 and Ki67 in predicting the prognosis of advanced NSCLC patients with EGFR mutation**. (**A**) There were no difference between CYFRA 21 − 1 positive and negative groups (median: 24.5vs20 months, *p* = 0.1370). (**B**)The difference between low and high Ki67 groups were also not observed (median: 26.5vs23.5 months, *p* = 0.5431)

Among 37 NSCLC patients with EGFR mutation, the median follow times was 21 months after TKIs treatment. survival analysis showed that patients with CYFRA 21 − 1 positive did not have shorter survival times than patients with CYFRA 21 − 1 negative (median: 24.5vs20 months, *p* = 0.1307; Fig. 6A) and Ki67 low and high groups (*p* = 0.5431; Fig. 6B).

The survival times of advanced NSCLC patients with EGFR mutation who have CYFRA 21 − 1 positive with Ki67 high were not significantly lower than patients with CYFRA 21 − 1 positive and Ki67 low (*p* = 0.1146; **Supplementary material 1 A**). Furthermore, in CYFRA 21 − 1 negative subgroup, the Ki67 also could not use as a predictive factor for patients with EGFR mutation (*p* = 0.4023, **Supplementary material 1B**).

### Univariate and multivariate cox analysis for OS in advanced NSCLC patients with wild-type EGFR

Univariate analysis showed that age (HR,1.035; 95%CI, 1.012 ~ 1.058; *p* = 0.002), ECOG score (HR, 2.316; 95% CI, 1.361 ~ 3.939; *p* = 0.002), and CYFRA 21 − 1 (HR, 2.538; 95% CI, 1.509 ~ 4.268; *p* < 0.001) were associated with OS in advanced NSCLC patients with wild-type EGFR (Table 3). However, gender, smoking history, CEA, SCC, TTF-1, CK-7, and Ki67 were not related to the prognosis of patients with wild-type EGFR in advanced NSCLC.

**Table 3:** Univariate and multivariate cox analysis for OS in advanced NSCLC patients with wild-type EGFR

Variables	Univariate analysis			Multivariate cox analysis		
	HR	95% CI	*p*	HR	95% CI	*p*
Gender	1.007	0.622 ~ 1.629	0.979	0.867	0.450 ~ 1.669	0.670
Age (years)	1.035	1.012 ~ 1.058	0.002	1.031	1.003 ~ 1.006	0.028
Histopathology	1.357	0.933 ~ 1.975	0.111	1.760	1.152 ~ 2.690	0.009
TNM stage	1.534	0.935 ~ 2.470	0.078			
ECOG score (> 2)	2.316	1.361 ~ 3.939	0.002			
Smoking history (ever)	0.988	0.632 ~ 1.546	0.958			
CEA (positive)	1.170	0.708 ~ 1.733	0.655			
SCC	0.859	0.306 ~ 2.415	0.774			
CYFRA21-1 (positive)	2.538	1.509 ~ 4.268	0.001	2.304	1.224 ~ 4.335	0.01
TTF-1 (positive)	0.711	0.404 ~ 1.251	0.237			
CK-7 (positive)	0.863	0.509 ~ 1.463	0.584			
Ki67 (High)	2.055	0.943 ~ 4.481	0.070	2.130	1.242 ~ 3.652	0.006

Note: Carcinoembryonic Antigen, CEA; Neuron Specific Enolase, NSE; Squamous Cell Carcinoma Antigen, SCC; Soluble Fragment of Cytokeratin 19, CYFRA 21 − 1; Thyroid transcription factor-1, TTF-1; cytokeratin-7, CK-7

Multivariate cox analysis showed that age (HR, 1.031; 95% CI, 1.003 ~ 1.006; *p* = 0.028), Histopathology (HR, 1.760; 95% CI, 1.152 ~ 2.690; *p* = 0.009), CYFRA 21 − 1 (HR, 2.304; 95% CI, 1.224 ~ 4.335; *p* = 0.01) and Ki67 (HR, 2.13; 95% CI, 1.242 ~ 3.652; *p* = 0.006) were independent risk factors for OS in those patients (Table 3). However, gender, history of smoking, ECOG score, CEA, SCC, TTF-1, and CK-7 were not related to the prognosis of patients with wild-type EGFR in advanced NSCLC.

## Discussion

Lung cancer is still one of the most malignant tumors in the world. At present, the main criteria for evaluating the prognosis of patients with NSCLC is still TNM stage [20]. However, the prognosis of patients who have the same tumor stage at the time of diagnosis, is still different, indicating that the criteria of NSCLC patient prognosis evaluation are required to update. Therefore, numerous studies are devoting to find suitable molecular markers that could early predict patients’ disease recurrence and metastasis.

Many studies have found that multiple factors were associated with the prognosis of patients with advanced NSCLC, especially patients with EGFR mutations [6, 21]. Yoshimura et al. demonstrated that the level of serum CEA at 1 month and CYFRA 21 − 1 at 4 months were significantly associated with OS. Moreover, the level of serum CYFRA 21 − 1 at 4 months was significantly correlated with improved OS according to multivariate analysis [22]. However, this study could not distinguish EGFR mutations and wild-type for patients. In our literature investigation, Filippo and colleagues demonstrated that CYFRA 21 − 1 might serve as a reliable prognostic biomarker for monitoring the effect of treatment by immune checkpoint inhibitors in advanced NSCLC patients, which may be associated with high tumor burden [23, 24]. Thus, our study further confirmed that CYFRA 21 − 1 could predict the survival of advanced NSCLC patients with wild-type EGFR or EGFR mutation. In addition, we also found that the level of serum CYFRA 21 − 1 and NSE are inversely correlated with the patient’s survival times (**Supplementary material 2**). However, due to the low goodness of fit, it might not be used to predict the prognosis of those patients.

Ki67 whose gene is located in the human genome 10q25 is mainly expressed in proliferating cells. It is one of the most widely used proliferation markers and many studies revealed that Ki67 in lung cancer tissues was closely related to the pathological characteristics of patients [15]. In addition, R Kremer et al’ study showed that Ki67 could distinguish patients with pleural effusion between lung cancer and benign inflammatory. It also reminded us that further research aimed at defining the best combination for marker analysis is needed [25]. However, many studies showed that there were no significant differences in clinical characteristics and patients with Ki67 high have shorter survival times than patients with Ki67 low [16, 26, 27]. Another study also indicated that Ki67 could predict the prognosis of patients with NSCLC by combing tumor metabolic and proliferative indices [28]. Our study shows that there are no significant differences in clinical features and OS between Ki67 low and high groups. A previous study demonstrated that Ki67 is mainly highly expressed in lung adenocarcinoma [29]. Since we could not divide squamous cell carcinoma and adenocarcinoma into two groups, it might cause Ki67 to serve as a better prognosis evaluation marker. Proliferating cells are usually accompanied by cell death and cell renewal, especially in tumor cells. Ki67 as an important marker for evaluating the proliferation activity of tumor cells and CYFRA 21 − 1 as a product released into the blood by the degradation of malignant epithelial cells could also evaluate the poor prognosis of the cancer [11]. Therefore, we proposed that the combination of CYFRA 21 − 1 and Ki67 could be used to predict the prognosis of advanced NSCLC patients with wild-type EGFR. The results of our study showed that the prognosis of patients with Ki67 high had a significantly adverse prognosis than patients with Ki67 low in the CYFRA 21 − 1 positive group. However, for patients with CYFRA 21 − 1 negative group, the difference in OS between Ki67 low and high groups was not observed. Besides, our study also found that Ki67 could not use as a risk stratification marker for patients with EGFR mutation in advanced NSCLC. Therefore, clinicians need to pay more attention to that advanced NSCLC wild-type EGFR patients with CYFRA21-1 positive and Ki67 high.

We were also aware of some limitations in our study. The results of single-center retrospective cohort studies are not generalizable to other populations. The sample size of patients that enrolled in the study was not large. The sample size needs to expand and distinguish sub-pathological types in the future. In conclusion, our study explored the clinical value of CYFRA 21 − 1 and Ki67 in predicting the prognosis of OS. If patients were CYFRA 21 − 1 positive and Ki67 high, it indicates that those patients have shorter survival times, which may provide new insights into EGFR wide-type NSCLC therapy and follow-up.

## Conclusion

In conclusion, elevated CYFRA21-1 was associated with worse OS in advanced patients with NSCLC while Ki67 was in patients with wild-type EGFR. In patients with wild-type EGFR, the combination of serum CYFRA21-1 and Ki67 had a better value to predict worse OS, and serum CYFRA21-1 positive and Ki67 high were independent prognostic factors for advanced NSCLC patients with wild-type EGFR. The prognostic significances of CYFRA21-1 and Ki67 in NSCLC varied with the EGFR mutation status.

## Electronic supplementary material

Below is the link to the electronic supplementary material.

Supplementary Material 1

Supplementary Material 2

## Data Availability

The original contributions are presented in this study are included in the article/Supplementary material. Requests to further inquiries can be directed to jszhengxq@163.com.

## References

[1] Siegel RL, Miller KD, Jemal A (2020). Cancer statistics, 2020. Cancer J Clin.

[2] Kocher F, Hilbe W, Seeber A, Pircher A, Schmid T, Greil R, Auberger J, Nevinny-Stickel M, Sterlacci W, Tzankov A (2015). Longitudinal analysis of 2293 NSCLC patients: a comprehensive study from the TYROL registry. Lung Cancer.

[3] Siegfried J, Gubish C, Rothstein M, Henry C, Stabile L (2012). Combining the multitargeted tyrosine kinase inhibitor vandetanib with the antiestrogen fulvestrant enhances its antitumor effect in non-small cell lung cancer. J Thorac Oncol.

[4] Cai Z (2016). Relationship between serum carcinoembryonic antigen level and epidermal growth factor receptor mutations with the influence on the prognosis of non-small-cell lung cancer patients. OncoTargets and therapy.

[5] Molina R, Marrades R, Augé J, Escudero J, Viñolas N, Reguart N, Ramirez J, Filella X, Molins L, Agustí A (2016). Assessment of a combined panel of six serum tumor markers for Lung Cancer. Am J Respir Crit Care Med.

[6] Woodard G, Jones K, Jablons D (2016). Lung Cancer Staging and Prognosis. Cancer Treat Res.

[7] Yu Z, Zhang G, Yang M, Zhang S, Zhao B, Shen G, Chai Y (2017). Systematic review of CYFRA 21 – 1 as a prognostic indicator and its predictive correlation with clinicopathological features in non-small cell lung Cancer: a meta-analysis. Oncotarget.

[8] Foa P, Fornier M, Miceli R, Seregni E, Santambrogio L, Nosotti M, Cataldo I, Sala M, Caldiera S, Bombardieri E (1999). Tumour markers CEA, NSE, SCC, TPA and CYFRA 21.1 in resectable non-small cell lung cancer. Anticancer Res.

[9] Niho S, Nishiwaki Y, Goto K, Ohmatsu H, Matsumoto T, Hojo F, Ohe Y, Kakinuma R, Kodama T (2000). Significance of serum pro-gastrin-releasing peptide as a predictor of relapse of small cell lung cancer: comparative evaluation with neuron-specific enolase and carcinoembryonic antigen. Lung Cancer.

[10] Dohmoto K, Hojo S, Fujita J, Ueda Y, Bandoh S, Yamaji Y, Ohtsuki Y, Dobashi N, Takahara J (2000). Mechanisms of the release of CYFRA21-1 in human lung cancer cell lines. Lung Cancer.

[11] Sheard M, Vojtesek B, Simickova M, Valik D (2002). Release of cytokeratin-18 and – 19 fragments (TPS and CYFRA 21 – 1) into the extracellular space during apoptosis. J Cell Biochem.

[12] Wang S, Ma P, Ma G, Lv Z, Wu F, Guo M, Li Y, Tan Q, Song S, Zhou E (2020). Value of serum tumor markers for predicting EGFR mutations and positive ALK expression in 1089 chinese non-small-cell lung cancer patients: a retrospective analysis. Eur J Cancer.

[13] Yao X, Gao H, Li C, Wu L, Bai J, Wang J, Li Y, Zhang Y (2017). Analysis of Ki67, HMGA1, MDM2, and RB expression in nonfunctioning pituitary adenomas. J Neurooncol.

[14] Lei B, Liu S, Qi W, Zhao Y, Li Y, Lin N, Xu X, Zhi C, Mei J, Yan Z (2013). PBK/TOPK expression in non-small-cell lung cancer: its correlation and prognostic significance with Ki67 and p53 expression. Histopathology.

[15] Ni J, Guo T, Li Y, Yang X, Li Y, Zou L, Chu L, Chu X, Li S, Ye L (2019). Patterns and risks of postoperative recurrence in completely resected EGFR-mutant non-small cell lung cancer: prognostic significance of routine immunohistochemical markers. Translational lung cancer research.

[16] Scagliotti G, Micela M, Gubetta L, Leonardo E, Cappia S, Borasio P, Pozzi E. Prognostic significance of Ki67 labelling in resected non small cell lung cancer.European journal of cancer1993(3):363–365. 10.1016/0959-8049(93)90387-u 8398336

[17] Ciancio N, Galasso M, Campisi R, Bivona L, Migliore M, Di Maria G (2012). Prognostic value of p53 and Ki67 expression in fiberoptic bronchial biopsies of patients with non small cell lung cancer. Multidisciplinary respiratory medicine.

[18] Li J, Yang F, Li X, Zhang M, Fu R, Yin X, Wang J (2017). Characteristics, survival, and risk factors of chinese young lung cancer patients: the experience from two institutions. Oncotarget.

[19] Rusch V, Chansky K, Kindler H, Nowak A, Pass H, Rice D, Shemanski L, Galateau-Sallé F, McCaughan B, Nakano T (2016). The IASLC Mesothelioma Staging Project: proposals for the M descriptors and for revision of the TNM Stage Groupings in the Forthcoming (Eighth) Edition of the TNM classification for Mesothelioma. J Thorac Oncol.

[20] Chilosi M, Murer B (2010). Mixed adenocarcinomas of the lung: place in new proposals in classification, mandatory for target therapy. Arch Pathol Lab Med.

[21] Wu Z, Dai Y, Chen L (2019). The prediction of epidermal growth factor receptor mutation and prognosis of EGFR tyrosine kinase inhibitor by serum ferritin in Advanced NSCLC. Cancer Manage Res.

[22] Yoshimura A, Uchino J, Hasegawa K, Tsuji T, Shiotsu S, Yuba T, Takumi C, Yamada T, Takayama K, Hiraoka N (2019). Carcinoembryonic antigen and CYFRA 21 – 1 responses as prognostic factors in advanced non-small cell lung cancer. Translational lung cancer research.

[23] Dall’Olio FG, Abbati F, Facchinetti F, Massucci M, Melotti B, Squadrilli A, Buti S, Formica F, Tiseo M, Ardizzoni A (2020). CEA and CYFRA 21 – 1 as prognostic biomarker and as a tool for treatment monitoring in advanced NSCLC treated with immune checkpoint inhibitors. Therapeutic Adv Med Oncol.

[24] Dall’Olio FG, Marabelle A, Caramella C, Garcia C, Aldea M, Chaput N, Robert C, Besse B (2022). Tumour burden and efficacy of immune-checkpoint inhibitors. Nat reviews Clin Oncol.

[25] Kremer R, Best L, Savulescu D, Gavish M, Nagler R (2010). Pleural fluid analysis of lung cancer vs benign inflammatory disease patients. Br J Cancer.

[26] Peng H, Tan X, Wang Y, Dai L, Liang G, Guo J, Chen M (2020). Clinical significance of Ki67 and circulating tumor cells with an epithelial-mesenchymal transition phenotype in non-small cell lung cancer. Am J translational Res.

[27] Yan S, Shun-Chang J, Li C, Jie L, Ya-Li L, Ling-Xiong W (2010). Topoisomerase II alpha expression and the benefit of adjuvant chemotherapy for postoperative patients with non-small cell lung cancer. BMC Cancer.

[28] Del Gobbo A, Pellegrinelli A, Gaudioso G, Castellani M, Zito Marino F, Franco R, Palleschi A, Nosotti M, Bosari S, Vaira V (2016). Analysis of NSCLC tumour heterogeneity, proliferative and 18F-FDG PET indices reveals Ki67 prognostic role in adenocarcinomas. Histopathology.

[29] Bozzetti C, Franciosi V, Crafa P, Carbognani P, Rusca M, Nizzoli R, Guazzi A, Naldi N, Cocconi G (2000). Biological variables in non-small cell lung cancer: comparison between immunocytochemical determination on fine needle aspirates from surgical specimens and immunohistochemical determination on tissue sections. Lung Cancer.

