# Supplementary figures and images for: Prognostic value of CYFRA 21 − 1 and Ki67 in advanced NSCLC patients with wild-type EGFR
Source: BMC Cancer. 2023 Mar 31;23:295. doi: 10.1186/s12885-023-10767-9 (PMC10064697; doi:10.1186/s12885-023-10767-9)

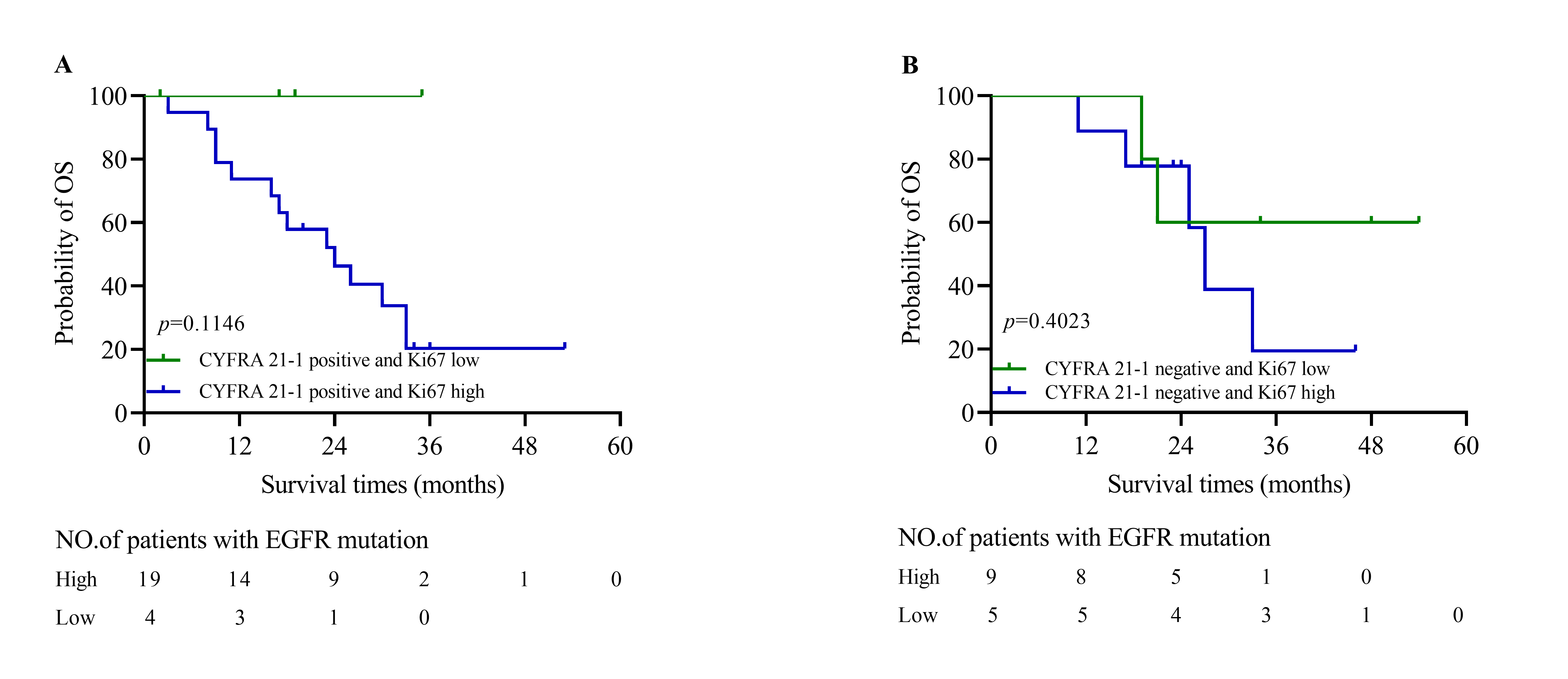

Supplement: Supplementary file 1 — Supplementary Material 1 [file 12885_2023_10767_MOESM1_ESM.tif]

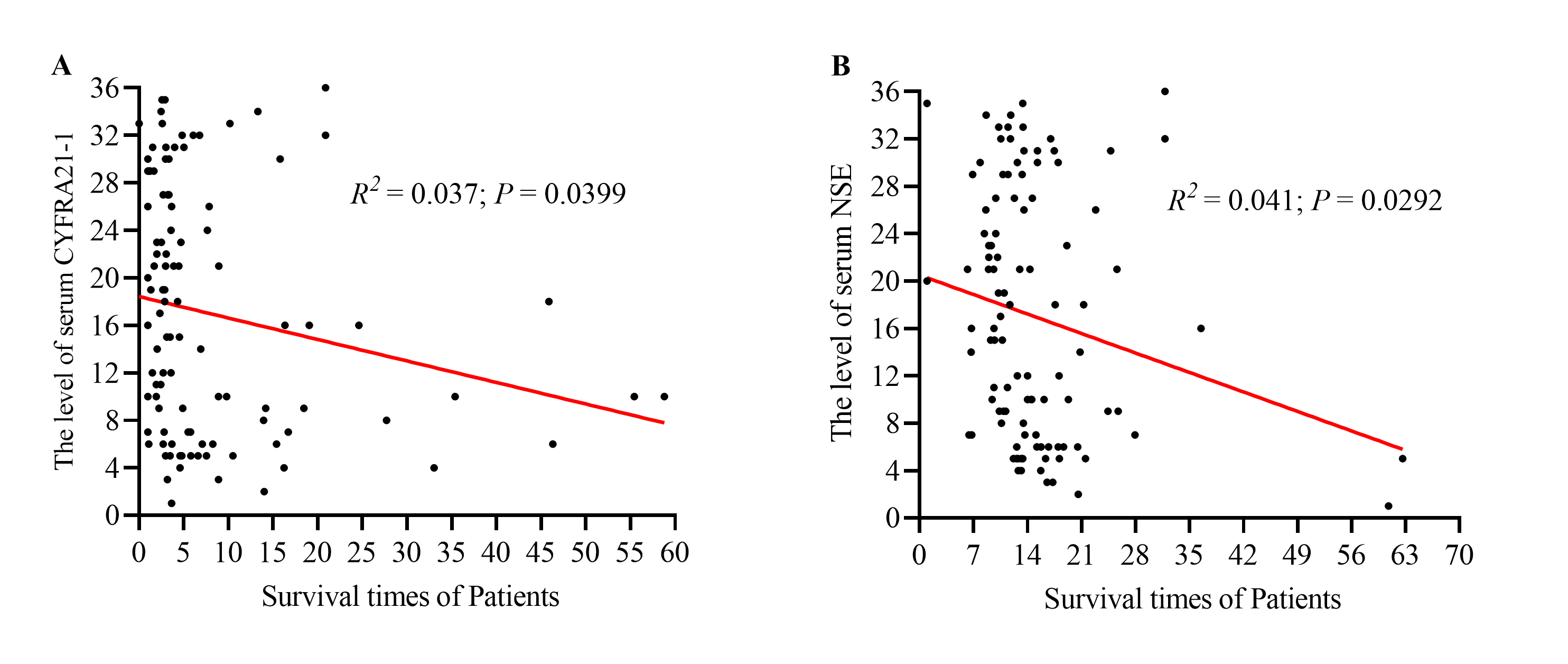

Supplement: Supplementary file 2 — Supplementary Material 2 [file 12885_2023_10767_MOESM2_ESM.tif]
